# Leveraging an Open-Access Digital Design Notebook for Graduate Biomedical Engineering Education in Nigeria

**DOI:** 10.1007/s43683-024-00136-8

**Published:** 2024-03-15

**Authors:** Padraic Casserly, Ademola Dare, Joy Onuh, Williams Baah, Ashley Taylor

**Affiliations:** 1 Rice University, Rice360 Institute for Global Health Technologies, Ibadan, Oyo Nigeria; 2 Department of Biomedical Engineering, University of Ibadan, Ibadan, Oyo Nigeria; 3 Rice University, Rice360 Institute for Global Health Technologies, Lagos, Lagos Nigeria; 4 Department of Biomedical Engineering and Mechanics, Virginia Tech, Blacksburg, VA USA

**Keywords:** Electronic notebook, Design notebook, e-portfolio, Google Sites, Biomedical engineering, Nigeria

## Abstract

Amidst the dual challenges of an eight-month university closure from nationwide public university strikes in Nigeria and the lingering impacts of the Covid-19 pandemic, we needed to innovate the delivery of BME graduate curriculum to ensure graduate students continued to progress in their studies. To ensure BME graduate students were engaging in team-based, clinician-identified engineering design challenges, we developed a digital design notebook (DDN) using Google Sites as an open-access, collaborative tool for scaffolding and documenting the engineering design process. Student design teams remotely uploaded digital content documenting their project work onto scaffolded DDNs created by program instructors. DDNs were purposefully designed to shepherd students through the design process such that each phase of the design process corresponded to an editable “page” of the DDN. Video lectures, learning resources, assignments, and other program information were embedded into the DDN for students to access throughout their design challenge. Project mentors and program instructors remotely monitored and assessed students’ work using the DDN. At the end of the design challenge, students effectively created an e-portfolio which showcased the work they conducted to build a biomedical prototype. Designing and implementing the DDN builds on previous research which demonstrates that “structured” design notebooks can be used as effective tools in engineering design and design thinking education. Our work also leverages educational frameworks for infusing engineering design into existing graduate biomedical engineering curriculum in Nigeria.

## Challenge Statement

Nigeria, upon realizing the crucial connection between engineering and national development, established several post-secondary engineering degree programs shortly after gaining independence in 1960. In the early 1960s, the excellent educational performance of engineering education in Nigeria was attributed to the availability of many engineering teachers who possessed industry knowledge [1]. However, in recent years, calls have increased to bridge the gap between engineering education and engineering industry in Nigeria [2], with the goal of fostering employability for engineering graduates.

Amidst these calls for advancing engineering education in Nigeria, two significant challenges since 2020 have necessitated the need to reimagine curriculum delivery within higher education institutions in Nigeria. First, starting in 2020, university closures due to the COVID-19 pandemic required educators to reimagine in-person learning experiences. Secondly, academic calendars in Nigeria have faced frequent disruptions due to strike actions by various unions, especially the Academic Staff Union of Universities (ASUU). Between 1999 and 2022, there were 13 strike actions with partial or full university closures, culminating to approximately 42 months of lost instructional time [3].

In 2022, we faced these dual dilemmas posing significant barriers to implementing a graduate internship program in biomedical engineering (BME) at a federal university in Nigeria, the University of Ibadan. Amidst an eight-month ASUU strike in 2022 and lingering impacts from the COVID-19 pandemic, we needed to innovate the delivery of BME graduate curriculum to ensure graduate students continued to progress in their studies.

## Novel Initiative

To ensure BME graduate students were engaging in authentic engineering design challenges amidst university closures, we developed a digital design notebook (DDN) using Google Sites as an open-access, collaborative tool for scaffolding and documenting the engineering design process. First, to source authentic biomedical engineering challenges, local clinicians were invited to submit proposals for graduate-level projects. Clinicians at a federal tertiary hospital in Ibadan, Nigeria, University College Hospital, shared challenges they face in their everyday clinical practice, along with ideas for appropriate project directions if applicable. BME faculty and staff at the university evaluated proposals from clinicians and selected projects that were most closely aligned with the BME graduate program learning objectives, which include:

1. Develop appropriate biomedical engineering solutions for clinicians in Nigeria by leveraging the engineering design process
2. Determine design specifications and constraints for an open-ended biomedical engineering design project
3. Generate design concepts for a biomedical engineering design project through brainstorming processes
4. Create a functional prototype of a biomedical engineering solution to meet design specifications
5. Collaborative effectively within a team to complete phases of the engineering design process

**Fig. 1:**
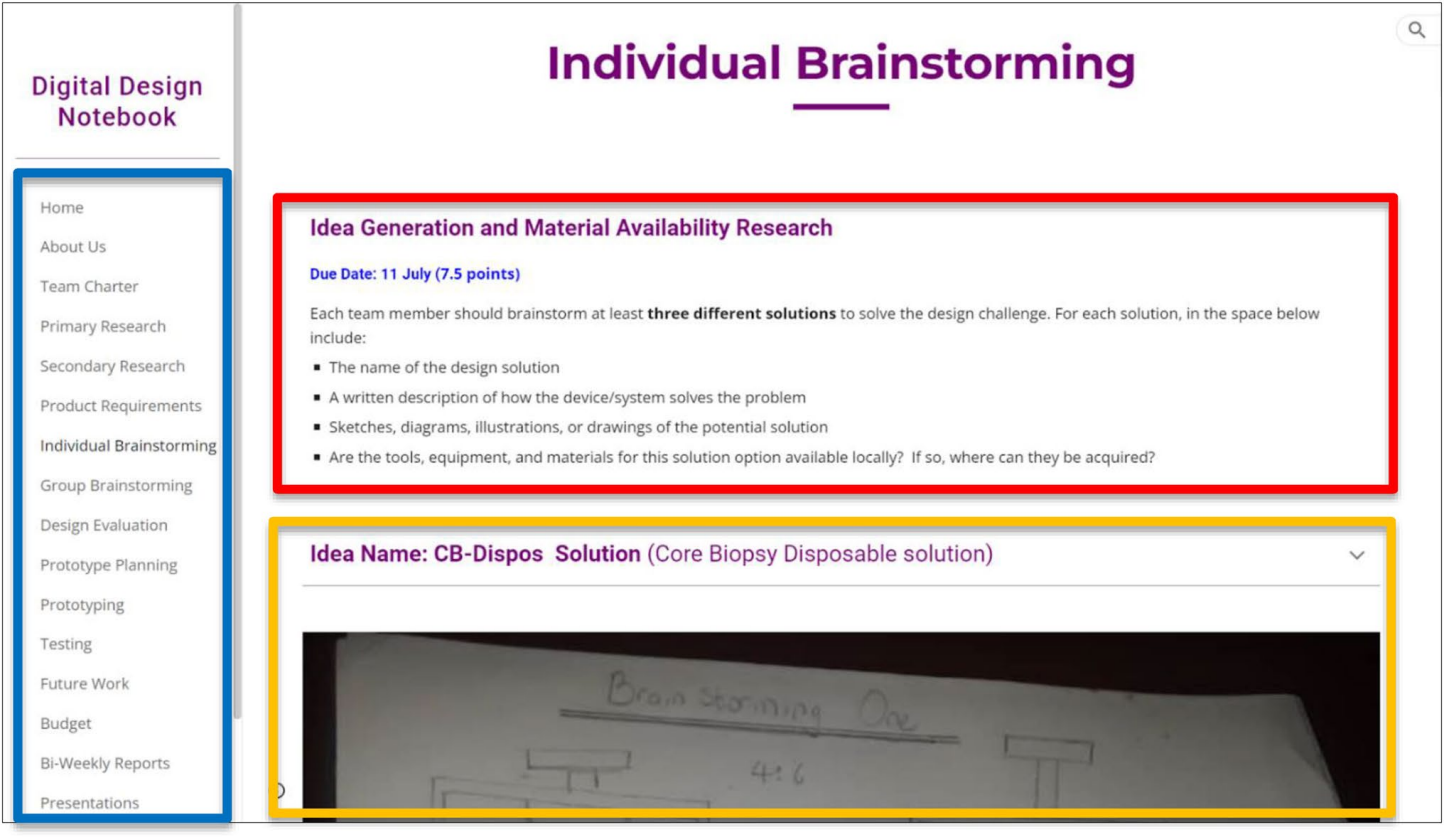
A screenshot from the “Individual Brainstorming” page of a design team’s digital design notebook (DDN). Each page of the team’s notebook (blue rectangle) corresponds to the different stages of the engineering design process. Assignments and learning material (red rectangle) were pre-loaded on to every page of each design team’s DDN before the course began. Content created by a student is seen at the bottom of the screenshot (orange rectangle). The idea name, “CB-Dispos,” represents a solution designed to enable core biopsy (i.e., “CB”) procedures using disposable (i.e., “Dispos”) instruments

For selected projects, clinicians served as primary mentors for the design projects. To scaffold students through each phase of the design process, beginning with needs-finding and concluding with low-fidelity prototyping and verification testing, the DDN was purposefully designed to host one phase of the design process (e.g., brainstorming) per “page” of the Google Site, as shown in Fig. 1. Through this design of the Google Site platform, the DDN incorporates two of Hogan and Pressley’s [4] definitions of scaffolding: (1) breaking content on the engineering design process into smaller tasks through the DDN, and (2) monitoring students’ progress in the engineering design process by having students reflect on progress and engaging project advisors in providing feedback through the DDN. To facilitate team-based design project work, nineteen students were divided into teams of two, with each team working on a distinct design project. One student worked alone on a design project, resulting in a total of ten different projects. Each team was assigned their own template DDN, preloaded with video lectures, assignments, deadlines, and instructional material for each stage of the engineering design process, and then tasked with completing DDN pages as they proceeded through their design project. All student contributions were tracked using Google Sites’ version control function. Google sites was chosen as the digital hosting platform due to its accessibility (i.e., free and open access) and seamless transferability across mobile devices, which is imperative for student users in Nigeria who may use a mobile device as their primary mechanism for internet access. Furthermore, prior research on the use of electronic notebooks to support team-based learning has suggested that students prefer open-access software applications for group recordkeeping over specialized electronic notebook products [5].

**Fig. 2:**
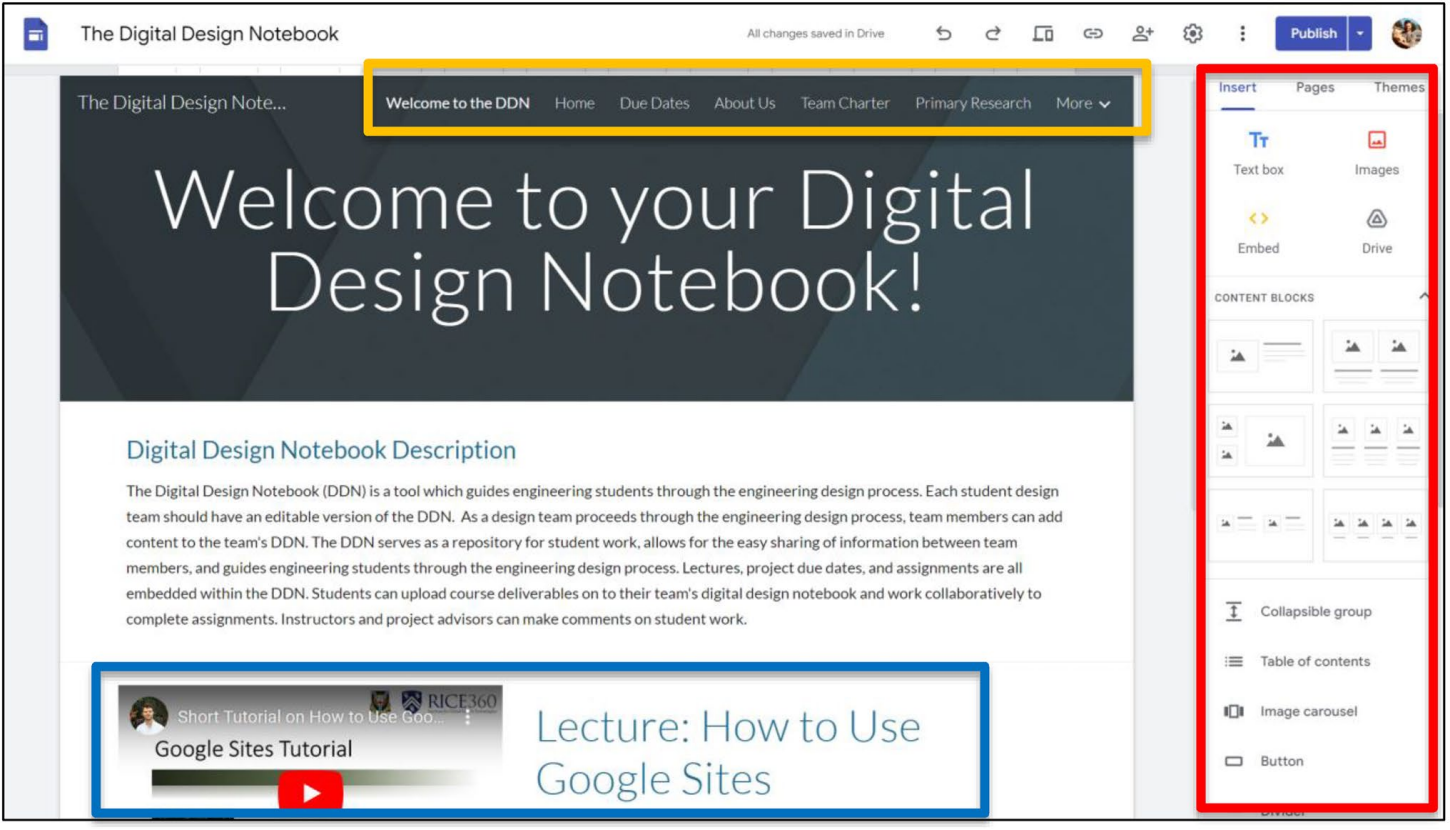
A screenshot from the editor view of the Google Sites interface. An authorized user can add new content or new pages to the DDN by selecting the content type from the righthand side of the interface (red rectangle). Students navigate through the different pages of the DDN using the navigation bar (orange rectangle) to view upcoming (and historical) assignments and deadlines and to access learning content. Video lectures (blue rectangle) on different course-related topics (e.g., building effective teams, design evaluation, etc.) were pre-loaded into the design notebooks before projects commenced

**Fig. 3:**
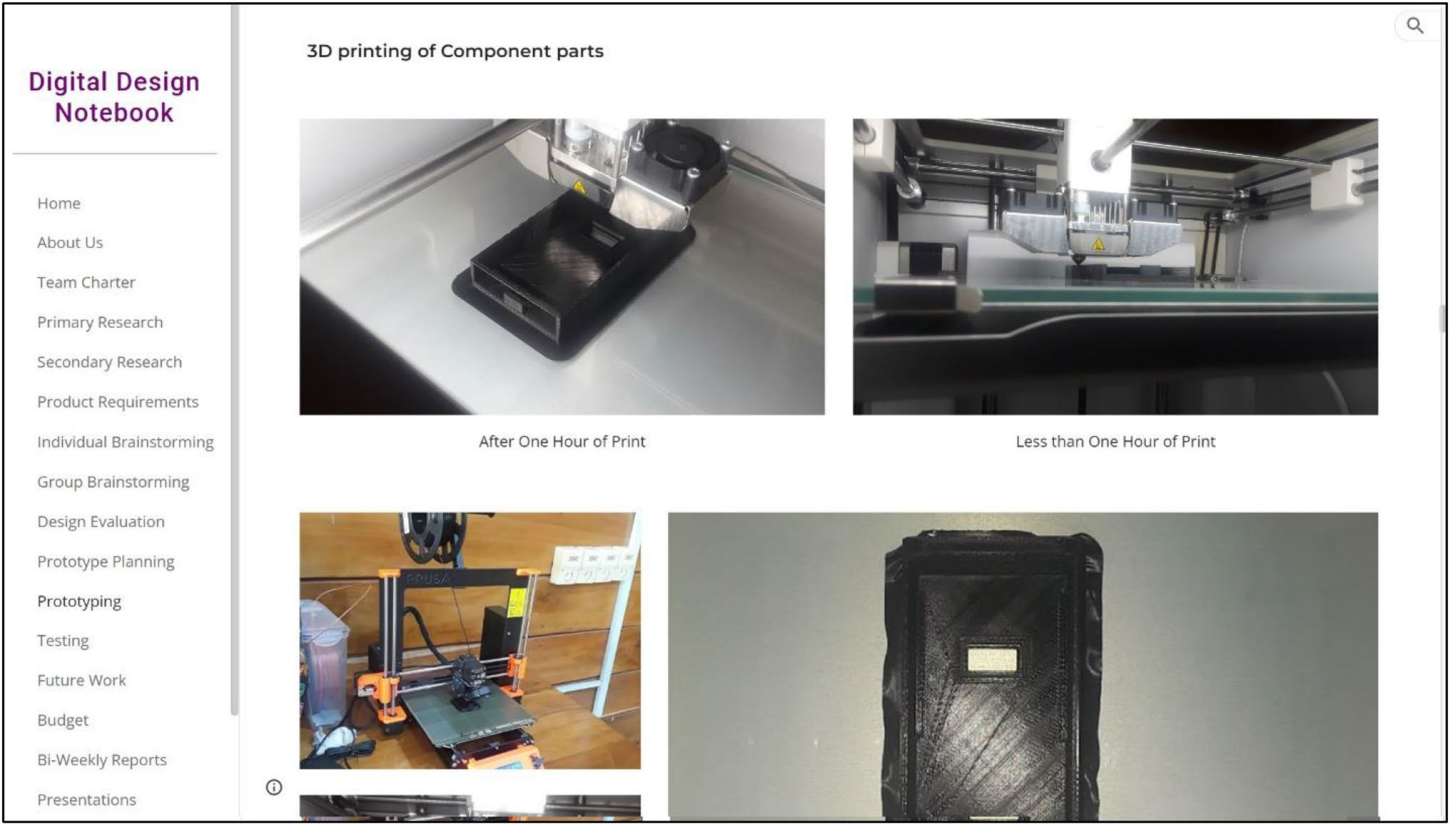
A screenshot from the “Prototyping” page of a design team’s DDN [6]. Students upload text, CAD models, pictures, video, and other digital artifacts onto their DDN

**Fig. 4:**
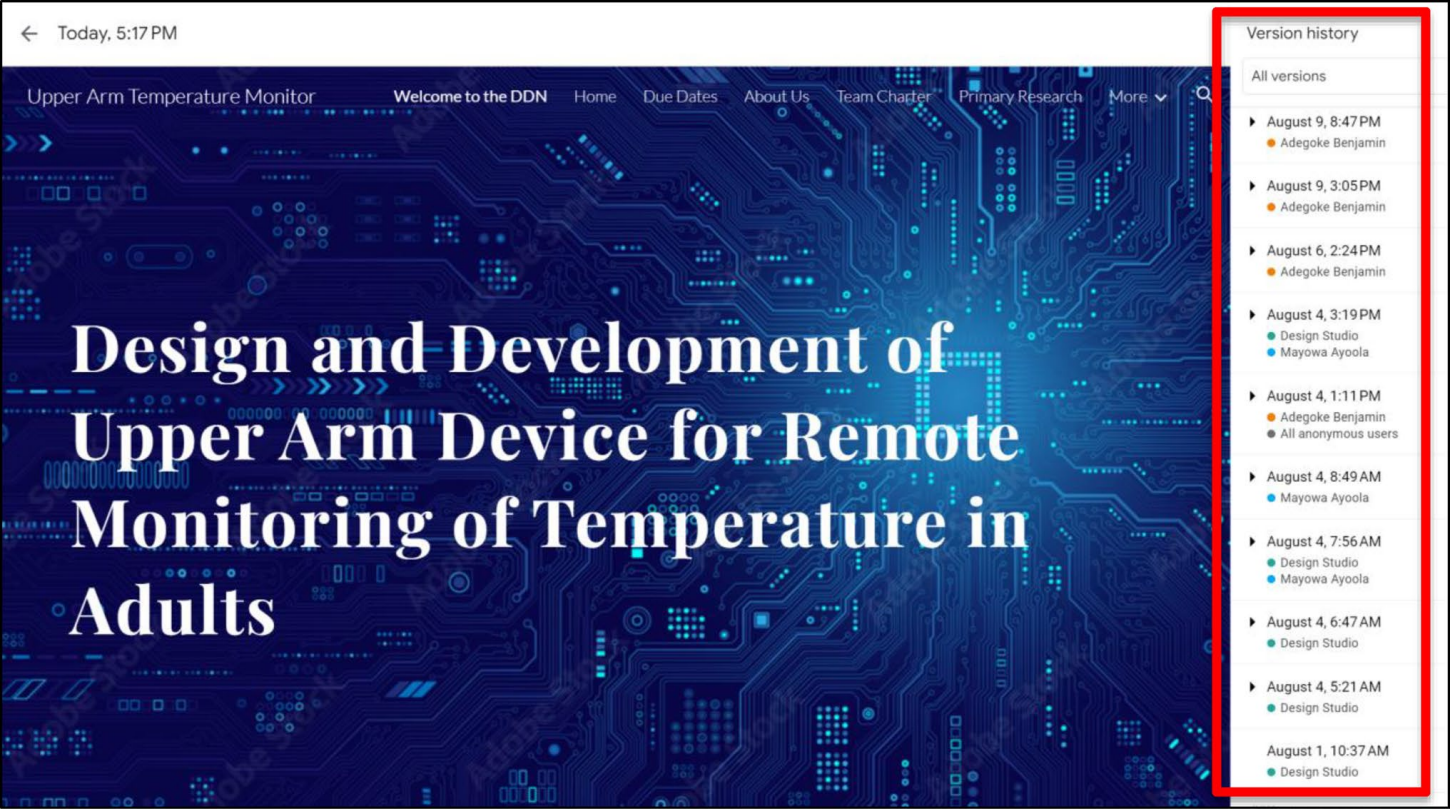
A screenshot of the version history view from a design team’s DDN. All authorized users can see what new content has been added to the DDN, when it was added, and by whom. They can also access past versions of the DDN using Google Sites’ version history feature (red rectangle)

As student teams proceeded through the engineering design process, they uploaded their work onto their team’s DDN, as illustrated in Figs. 1, 2, and 3. Program administrators and design team advisors were able to track the progress of each design team remotely. Team advisors added comments and feedback to students’ work directly within the DDN. Advisors also monitored the individual contributions made by each team member by leveraging the version history feature of Google Sites, as shown in Fig. 4.

## Reflection

The DDN, hosted on Google Sites, provided an invaluable platform for adapting the BME graduate education curriculum at the University of Ibadan during the ASUU strike and COVID-19 pandemic. Even after these challenges subsided, the DDN continues to be used by BME graduate students at the university to structure and document their project work. Our use of the DDN to embed authentic problem-based learning experiences within the broader BME graduate curriculum aligns with recommendations from the 2019 BME Education Summit, enabling graduate students to engage in two critical BME curricular elements [6]: (1) open-ended design projects that address unmet health needs in Nigeria and (2) culminating design experiences that promote interdisciplinary collaboration. Ultimately, our use of the DDN supports the professional preparedness of BME graduates in Nigeria.

Our work augments previous literature which suggests design notebooks serve many useful functions in scaffolding and assessing the engineering design process. Through our pilot assessment of students’ experiences with the DDN, we learned that students used the DDN to organize project work, keep notes, sketch out ideas, and retrieve information. Clinicians, who served as project mentors, also leveraged the DDN to monitor students’ progress and provide technical feedback to teams. Our findings align with prior research which found that student maintenance of design notebooks supports project planning, improves record keeping, and compliments the creative process as students generate and evaluate ideas for design projects [7, 8].

Similarly, our work builds on the research of Bergsman [9] who demonstrated that “structured” engineering design notebooks can be used as effective tools in engineering design and design thinking education [9]. Bergsman developed a physical, “structured” engineering design notebook with pre-defined prompts to scaffold students’ design thinking mindset and support their navigation of design thinking techniques. Bergsman drew three primary conclusions from the research that also illuminate our findings: (1) engineering design courses benefit by including instructional “scaffolds” for the engineering design process and project management; (2) a structured design notebook provides support for robust documentation practices; and (3) students prefer to submit design notebook artifacts digitally [10]. We expand Bergsman’s innovative work by extending the scaffolded, structured design notebook to an open-access, digital platform and embedding instructional content directly into the learning platform.

The design and implementation of the DDN also leverages Denson and Lammi’s [10] framework for infusing engineering design into existing learning environments [10]. The framework, which synthesizes existing literature on engineering design experiences, urges educators to consider four key principles when integrating engineering design into learning environments. Though their framework [10] describes integration of engineering design into high school learning environments, we adapted these four principles to our context of a higher education environment in Nigeria, as described below:

1. *Situate design within the curriculum* Denson and Lammi (2014) urge educators to situate engineering design curriculum within existing institutional curricula. As a form of inquiry-based instruction, the engineering design process can complement existing curriculum while remaining relevant to existing, context-specific standards for education [11–13]. We applied this principle by integrating the DDN into the university's BME internship, which is a core component of the existing BME graduate curriculum.
2. *Select appropriate design challenges* The framework leverages Householder and Hailey’s [14] definition of design challenges as “ill-structured problems that may be approached and resolved using strategies and approaches commonly considered to be engineering practices” (p. 2). Design challenges should harness student motivation by being authentic (i.e., connected to real-world problems) and open-ended (i.e., providing opportunities for student agency in defining the solution path). We underpinned our implementation of the DDN by partnering with local clinicians at the local tertiary hospital to select authentic biomedical engineering challenges. Ultimately, selecting relevant BME challenges from the community fostered stronger connections between the BME graduate program and the local innovation ecosystem.
3. *Sequence the design experience* By nature, engineering design challenges are often ill-structured. The engineering design process provides an iterative, structured process to support systematic problem solving, which Carr and Strobel [15] describe as points on a continuum. Effective design education, therefore, must provide a clear sequential pathway for students to follow through the engineering design process [11]. The DDN provided highly structured sequencing for students built into the platform, providing a digital, sequential pathway of the engineering design process.
4. *Assess students’ experience* Much debate exists on how to best develop and assess students’ engineering design experience [10, 16, 17]. Design experiences are challenging to assess due to the open-ended nature of design challenges [10] as well as the iterative nature of the engineering design process [17, 18]. Engineering notebooks are one assessment tool that provides a lens into a design team’s group work [19]. To support formative assessment, the DDN provided rubrics embedded into the digital platform as a practical tool for providing feedback to students.

To assess the impact of the DDN on student learning, we initially leveraged two primary data sources: (1) reflections from the Biomedical Engineering Program Head comparing the DDN to previous methods of assessment, and (2) pilot mixed-methods assessment data using pre- and post-program surveys administered to students that included the Mamaril et al. [20] Engineering Self Efficacy Scale and open-ended questions to provide the opportunity for thematic analysis of qualitative data. Reflections from the Program Head, a co-author of this work, indicate that the DDN is a significant improvement to “progress reports,” which have historically been used by the department to assess students’ learning. The Head shared that the DDN provides more detailed insight into students’ work than the previously used progress reports, providing an opportunity for faculty to view students weekly progress through the DDN and provide responsive feedback. Additionally, the instructor team more broadly saw an improvement in the quality of students’ design projects and overall engagement with the program. Moving forward, the Head will survey all department project advisors to more fully evaluate their experiences with assessing students’ learning through the DDN as compared to the previous approach of progress reports.

Through our pilot mixed-methods survey assessment data, we learned that students used the DDN as a practical collaboration tool to sequence and track their progress. However, our pilot data was significantly limited, as only six of the 19 graduate students completed both the pre- and post- program surveys. Additionally, although students shared positive feedback about their experiences utilizing the DDN, their responses to open-ended reflection questions lacked depth. Combined, our insufficient pilot assessment data suggest a major opportunity to improve our plan for assessing student learning by redesigning our assessment plan as follows:

1. Expand survey to include student reflection prompts on the Engineering Habits of Mind
  Our utilization of the Mamaril et al. [20] Engineering Self-Efficacy Scale was a strength of our pilot assessment and will serve as a cornerstone of our future pre- and post-program survey design. However, we learned that our open-ended reflection questions require a significant overhaul to illicit meaningful reflections from students on their engineering design process learning. We reevaluated the alignment of our assessment plan with the learning outcomes for the program, which broadly center on teamwork and the engineering design process- a ‘signature pedagogy’ for biomedical engineering [21, 22]. Moving forward, our pre- and post-program surveys will include both the Mamaril et al. [19] Engineering Self Efficacy Scale and six reflection prompts for student rooted in each construct of the Engineering Habits of Mind (EHoM): systems-thinking, problem-finding, visualizing, improving, creative problem solving, and adapting [21]. By leveraging the EHoM as a framework to guide student reflection prompts, our goal is to assess in a more nuanced way how our students’ thinking about and actions throughout the engineering design process evolve as they use the DDN.
2. Triangulate students’ self-reported assessment data with perspectives from clinical mentors and instructors
  A major strength of the pilot implementation of the DDN was the strong engagement from instructors and clinical mentors; however, our pilot assessment failed to incorporate instructors and clinical mentors meaningfully. Therefore, a significant opportunity for improving our assessment plan is to incorporate all program stakeholders in the assessment process. Moving forward, our assessment data collection will include pre- and post-program surveys to all instructors and clinical mentors interacting with students through the DDN. In alignment with our student survey expansion noted above, we will develop open-ended reflection prompts based on the six constructs of the EHoM as observed in students’ work by instructors and clinical mentors. Our expanded assessment plan, which will include all stakeholders interacting with the DDN, will enable us to triangulate data in order to more fully assess the impact of the DDN on student learning.
3. Refine implementation of data collection strategies to ensure students complete both pre- and post- surveys
  Finally, a major limitation of our pilot data was the attrition rate on our post-program survey. In collaboration with the Biomedical Engineering Education Department Head, we will mitigate this challenge in our future assessment plan by incorporating data collection into existing departmental student assessments. Embedding the DDN student learning assessment within existing program surveys will support institutionalization of the DDN in addition to improving response rates, avoiding survey fatigue for student participants, and enabling meaningful statistical analysis of pre- and post-program responses.

As we work to develop a shared understanding of best practices in graduate BME education, we must consider how to position curricula to remain adaptive amidst emerging challenges. We share our experience using a DDN as an open-access tool for integrating engineering design into graduate BME curriculum. This tool arose from the urgent need to adapt teaching strategies during simultaneous challenges, including a national strike resulting in public university closures and the COVID-19 pandemic. For educators encountering their own challenges which necessitate curricular innovation, we offer our experience as a starting point for a broader conversation on leveraging open-access tools to support a more resilient BME education community. There exists a promising opportunity to support curricular innovation in graduate BME programs, particularly for innovation and design education [20]. Our current work contributes to this curricular innovation and to the broader literature in biomedical engineering education by describing the design, implementation, and assessment of the digital design notebook to scaffold and assess the engineering design process within the underexplored context of a graduate BME program in Nigeria.

For readers interested in viewing a copy of the digital design notebook used in this course and request your own copy, visit https://sites.google.com/view/digital-design-notebook.

## Data Availability

Not applicable.
